# Radiologic analysis of large vestibular schwannoma position on surgical outcomes

**DOI:** 10.1007/s11060-026-05639-8

**Published:** 2026-06-03

**Authors:** Hayley A. Granberg, Alireza Zonnour, Gabriela S. Navarro-Parga, Kenneth Thomas, Michael G. Brandel, Krish Suresh, Rick Friedman, Marc Schwartz

**Affiliations:** 1 Rocky Vista University College of Osteopathic Medicine, Parker, CO USA; 2 Department of Otolaryngology, University of California San Diego, San Diego, CA USA; 3 Centro Universitario de Ciencias de la Salud, Universidad de Guadalajara, Guadalajara, Mexico; 4 Department of Neurological Surgery, University of California San Diego, San Diego, CA USA

**Keywords:** Vestibular schwannoma, Acoustic neuroma, Brainstem compression, Internal auditory canal, Tumor extension, Outcomes

## Abstract

**Background:**

Large vestibular schwannomas (VS) often compress the brainstem and differ in their relation to the internal auditory canal (IAC); the significance of these radiographic features on postoperative outcomes remains unclear. This study quantifies the impact of brainstem compression (BSC) and position relative to the IAC on surgical outcomes in VS.

**Methods:**

We retrospectively identified 116 patients with sporadic unilateral VS ≥ 3 centimeters (2017–2022). Neurofibromatosis 2 cases were excluded. BSC was quantified with MRI T1 post-contrast axial images as the perpendicular distance from the brainstem-cerebellum to the point of maximal compression. Anterior and posterior IAC extension were measured relative to a line bisecting the IAC from the porus to fundus. Outcomes included postoperative facial nerve (FN) function, extent of resection (EOR), and length of stay (LOS).

**Results:**

Greater anterior extension was associated with decreased EOR in univariate analysis (OR = 1.12, *p* = 0.03), but not after controlling for tumor size and age (OR = 1.09, *p* = 0.158). Greater BSC was associated with worse FN function at 2–3 weeks postoperatively on univariate (OR = 1.08, *p* = 0.036) and approached significance on multivariate analysis (OR = 1.07, *p* = 0.08). Posterior extension was associated with increased LOS in univariate (β = 217.57 min, *p* = 0.024), but not multivariate analysis. Neither anterior extension nor BSC were associated with LOS. Older age correlated with a lower rate of GTR and longer LOS in multivariate analysis (EOR: OR = 1.05, *p* = 0.003; LOS: β = 79.84 min, *p* = 0.026).

**Conclusion:**

BSC is a radiographic feature that may be associated with short-term FN outcomes. EOR and LOS do not appear to be influenced by anterior or posterior extension, respectively, but rather appear to be confounded by age. These findings may inform individualized preoperative counseling and surgical planning for large VS.

## Introduction

Vestibular schwannoma (VS) is a slow growing benign tumor commonly arising from the vestibulocochlear nerve that accounts for ~ 8% of all intracranial tumors [1, 2]. These tumors are often sporadic and unilateral, originating in the internal auditory canal (IAC) and extending into the CPA [2]. Management of VS includes observation, stereotactic radiosurgery, or surgical removal [3–7]. Surgical resection is most often employed in patients with large VS [2].

There is substantial interest in identifying clinical predictors of surgical outcomes in VS. To date, extent of resection (EOR) and tumor size are consistently reported to influence functional outcomes [2, 7–9], with two recent studies indicating that ventral extension of the tumor may also play a significant role [10, 11]. Furthermore, tumor morphological characteristics such as brainstem compression (BSC) may be a significant predictor of facial nerve (FN) function [11].

We aimed to quantitatively assess the impact of BSC and position relative to the IAC on surgical outcomes in large VS. We hypothesized that increasing axial extension in the anterior and posterior direction relative to the IAC, and increasing BSC, would be associated with worse FN outcome (FNO), lower GTR rates, and longer hospital LOS.

## Methods

### Patient selection

A retrospective review of a prospectively maintained database of all patients with large VS at a single tertiary care center in the United States between 2017 and 2022 was performed. Study protocol approval was obtained from the institutional review board from the institution, and all patients gave informed consent for research. Patients were included for analysis if they had a sporadic, unilateral VS ≥ 3 centimeters (cm) in any dimension diagnosed through magnetic resonance imaging (MRI). Patients with tumor pathology other than VS, neurofibromatosis 2 (NF2), and prior surgical resection or radiation that may have affected the brainstem tumor interface were excluded.

### Data extraction

Age, sex, height, weight, race, ethnicity, smoking status, tumor size, EOR, and postoperative FN function data were collected. FN function was reported using House-Brackman (HB) grading system. EOR was based on intraoperative findings. GTR was designated when no gross residual tumor. NTR was defined as the preservation of a thin layer of residual tumor measuring < 1 cm in linear extent as determined by the senior Neurosurgeon. Residual tumor was left at sites of maximal adherence, most commonly along the facial nerve, but also along the brainstem or any other neurovascular or anatomical structure where the tumor was particularly adherent and removal risked neurologic dysfunction. STR was designated when any residual tumor exceeding 1 cm in linear extent was left behind intraoperatively.

### Radiographic analysis

**Fig. 1:**
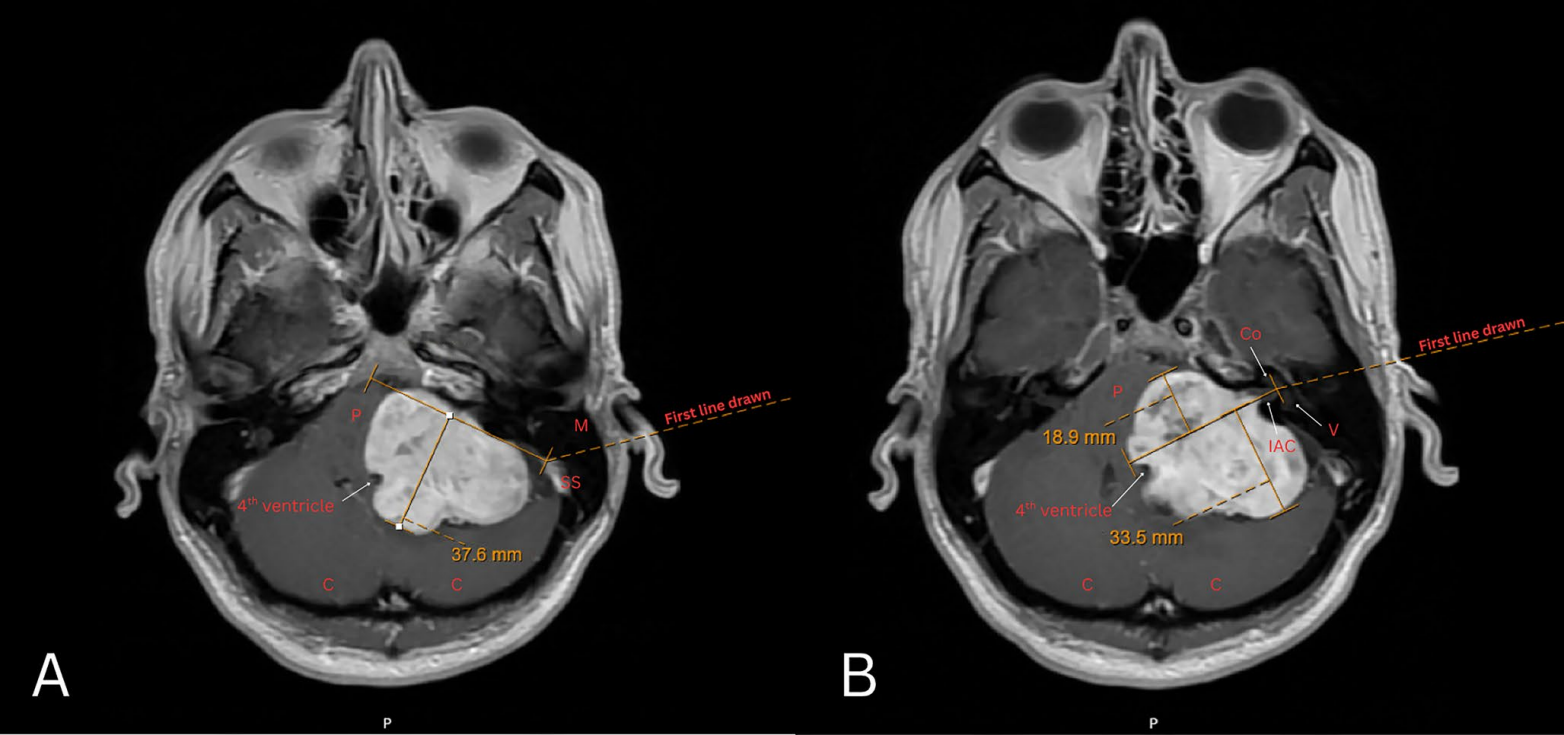
Annotated T1 MRI illustrating example measurements of BSC (**A**), anterior and posterior extension (**B**). Integers with arrows pointing to the associated structures are as follows: P – pons; C – cerebellum; Co – cochlea; IAC – internal auditory canal; M – mastoid; SS – sigmoid sinus; V – vestibule

MRI axial T1-weighted post-contrast sequences were used for measurement. To measure BSC, a line was drawn from the anteromedial point along the brainstem where there was no compression to the posterolateral point along the cerebellum where there was no compression. Then, a second perpendicular line was drawn to the medial most edge of the tumor to measure the extent of maximal BSC (Fig. 1A). To measure the degree of anterior-posterior extension of a tumor relative to the IAC, a baseline was drawn that bisected the IAC, from the mid-section of the fundus through the porous acousticus. Anterior and posterior tumor extension was determined by drawing perpendicular lines from this baseline to the most anterior or posterior extent of the tumor (Fig. 1B).

### Statistical analysis

Continuous variables were summarized with medians, interquartile ranges (IQRs), and ranges. Categorical features were summarized with frequency counts and percentages. Univariate logistic regression was performed to assess associations between patient characteristics and binary outcomes including EOR (GTR versus non-GTR), good FN function (HB I–II) versus poor FN function (HB III–VI) at multiple timepoints (postoperative day 1, 2–3 weeks postoperatively, and at latest follow-up) (Table 1). Resection rates were grouped as either GTR or non-GTR for binary logistic regression for increased statistical power as the NTR group only had four patients. Continuous linear regression was used to model hospital LOS in minutes. Variables with *p* < 0.05 in univariate models were considered candidate predictors for multivariate analysis. Multivariate models were manually constructed based on these predictors, clinical relevance, best fit using Akaike Information Criterion (AIC), and avoidance of multicollinearity. A predictor-to-event ratio of at least 10:1 for each variable was met to prevent overfitting the models. *P* values < 0.05 were considered statistically significant. Odds ratios (ORs) and 95% confidence intervals (CIs) were reported for binary outcomes; beta (β) estimates and 95% CIs were reported for the continuous LOS outcome. All confidence intervals were reported at 95%. Statistical analyses were performed using the coding language R in RStudio version 2024.12.1.563 (Posit Software, PBC, Boston, MA).

**Table 1:** Postoperative FN function N (%)

HB grade	Postoperative Interval		
	POD1	2–3 weeks	Latest Follow-up*
I	69 (59.5)	63 (54.7)	47 (59.5)
II	20 (17.2)	20 (17.2)	12 (15.2)
III	15 (12.9)	5 (4.3)	8 (0.1)
VI	6 (5.2)	7 (6)	3 (0.03)
V	2 (1.7)	7 (6)	3 (0.03)
VI	4 (3.4)	14 (12.1)	6 (0.08)
NR			37 (46.8)

Legend: FN – facial nerve; HB – House Brackman; POD1 – postoperative day 1; NR – not reported. *Note: Among the patients with available admit and latest follow-up dates, median follow-up was 13.2 months (interquartile range 4.4–26.6; range 0.3–81.4 months)

**Table 2:** Summary of patient demographics

*N* = 116	*N* (%)	Median (IQR)	Mean (Range)
Demographics			
Age (years)	116	39.5 (32–52)	42 (9–77)
Sex (F/M)	65/51 (56%/44%)		
Weight (pounds)		167.4 (142.2-197.5)	177.1 (83–360)
Height (inches)		66 (64–69)	66.44 (54–75)
BMI (kg/m^2^)		26.89 (23.32–30.52)	28.10 (15.14–59.9)
Underweight < 18.5	5 (4.3%)		
Normal 18.5–24.9	37 (31.9%)		
Overweight 25-29.9	39 (33.6%)		
Obese > 30	35 (30.2%)		
Race			
AIAN	1 (0.9%)		
Asian	13 (11.2%)		
Black or AA	2 (1.7%)		
NHOPI	0 (0)		
Other or Mixed	18 (15.5%)		
Unknown	2 (1.7%)		
White	80 (69%)		
Ethnicity			
Non-Hispanic	94 (81%)		
Hispanic	13 (11.2%)		
Hispanic/Latino	9 (7.8%)		
Smoking Status			
Current Smoker	3 (2.6%)		
Former Smoker	19 (16.4%)		
Never Smoker	72 (62%)		
Never Assessed	22 (19%)		
Tumor Characteristics			
MLD on MRI (mm)		37.5 (33.00–42.00)	38.43 (12.40–80.00)
BSC (mm)		25.00 (21.00-29.32)	25.24 (12.00-43.30)
Ant ext to IAC (mm)		10.20 (8.08–14.13)	11.03 (4.00–23.00)
Post ext to IAC (mm)		22.15 (19.18–25.15)	22.46 (9.00-39.50)
Extent of Resection			
GTR	49 (42.2%)		
NTR	4 (3.4%)		
STR	63 (54.3%)		
Surgical Approach			
TL	112 (96.5%)		
RS	4 (3.5%)		
LOS (minutes)		4728 (3718–5994)	6181 (982-48004)

Legend: BMI – body mass index; F – female; M – male; AIAN – American Indian or Alaska Native; AA – African American; NHOPI – Native Hawaiian or other Pacific Islander; MLD – maximum linear dimension; MRI – magnetic resonance imaging; mm – millimeters; BSC – brainstem compression; Ant ext – anterior extension relative to the IAC; IAC – internal auditory canal; Post ext – posterior extension relative to the IAC; GTR – gross total resection; NTR – near total resection; STR – subtotal resection; LOS – hospital length of stay

## Results

**Fig. 2:**
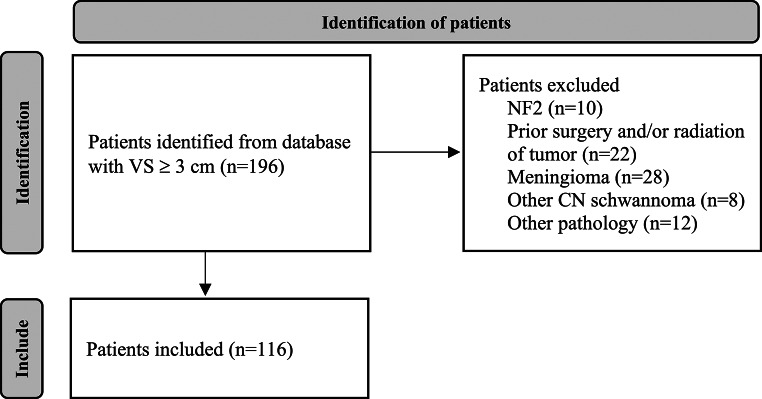
Flowchart demonstrating patients identified in database for inclusion. Legend: VS – vestibular schwannoma; NF2 – neurofibromatosis 2; CN – cranial nerve. *Other pathology includes adjacent fluid collection (likely arachnoid cyst) causing brainstem compression (*n* = 1), cerebral cavernoma (*n* = 1), epidermoid cyst (*n* = 1), glomus jugulare (*n* = 2); multiple complex tumors (*n* = 2), chondrosarcoma (*n* = 1), hemangioblastoma (*n* = 1), unable to access patients chart (*n* = 3)

One hundred ninety-six patients were initially identified. Of these, 80 were excluded due to not meeting inclusion criteria (Fig. 2). One-hundred sixteen patients had VS pathology ≥ 3 cm and were included in the final analysis. 56% (*n* = 65) patients were female; 44% (*n* = 51) male. The median age at the time of surgery was 39.5 years (IQR 32–52 years); mean age was 42 years (range 9–77 years). The median age for GTR patients was 36 years (*n* = 49) whereas the median age for non-GTR patients was 41 (*n* = 67). Median tumor maximum linear dimension (MLD) was 37.5 mm (IQR 33.00–42.00); mean tumor MLD was 38.43 mm (range 12.40–80.00). Median BSC was 25.00 mm (IQR 21.00–29.32); mean BSC was 25.24 mm (range 12.00–43.30). Median posterior extension was 22.15 mm (IQR 19.18–25.15); mean posterior extension was 22.46 mm (range 9.00–39.50). Median anterior extension was 10.20 mm (IQR 8.08–14.13); mean anterior extension was 11.03 mm (range 4.00–23.00). Median LOS: 3.28 days (IQR 2.58–4.16); mean LOS: 4.29 days (range 0.68–33.34). Further demographics are listed in Table 2.

### EOR Outcomes

Forty-nine patients (42.2%) had GTR, 63 (54.3%) had STR, and 4 (3.4%) had NTR. Greater anterior extension was significantly associated with a decreased likelihood of achieving a GTR in univariate analysis—for each 1 mm increase in anterior extension, the odds of having a worse resection (non-GTR) increased by 12% (OR = 1.12, 95% CI 1.015–1.256, *p* = 0.0295) (Table 3). This relationship remained significant in a partially adjusted multivariate model after controlling for posterior extension and BSC (OR = 1.13, 95% CI 1.019–1.267, *p* = 0.0256); however, this relationship lost significance in a fully adjusted multivariate analysis additionally adjusting for tumor size (OR = 1.12, 95% CI 0.997–1.254, *p* = 0.059) and age (OR = 1.09, 95% CI 0.968–1.233, *p* = 0.158) (Table 4). BSC and posterior extension did not impact EOR in univariate or multivariate analyses (*p* > 0.05). Older age at the time of surgery was significantly associated with higher likelihood of achieving a lower EOR (i.e., STR or NTR) in univariate (OR = 1.05, 95% CI 1.02–1.08, *p* = 0.002) and multivariate models (OR = 1.05, 95% CI 1.02–1.08, *p* = 0.003), meaning that each additional year of age increased the odds of non-GTR by ~ 5%. Sex, race, BMI, ethnicity, smoking status were not associated with EOR in univariate or multivariate analyses (*p* > 0.05) (Tables 3 and 4).

**Table 3:** Single predictor analysis of EOR, LOS, FN function

*N* = 116	EOR (ref GTR)			LOS			FN (POD1)			FN (2–3 weeks postop)			FN (latest)		
	OR	95% CI	*P*	Coefficient (β) (min)	95% CI	*P*	OR	95% CI	*P*	OR	95% CI	*P*	OR	95% CI	*P*
Demographics															
Age	1.05	1.02–1.08	0.002*	89.73	20.85–158.62	0.011*	1	0.97–1.03	0.9823	1.01	0.98–1.04	0.408	1	0.97–1.04	0.796
Sex															
Female	*Ref*	*Ref*	*Ref*	*Ref*	*Ref*	*Ref*	*Ref*	*Ref*	*Ref*	*Ref*	*Ref*	*Ref*	*Ref*	*Ref*	*Ref*
Male	1.08	0.51–2.28	0.837	1178.36	-886.2–3242.75	0.261	1.51	0.64-–3.63	0.348	1.82	0.81–4.15	0.15	3.62	1.28–11.02	0.018
BMI															
Non-obese	*Ref*	*Ref*	*Ref*	*Ref*	*Ref*	*Ref*	*Ref*	*Ref*	*Ref*	*Ref*	*Ref*	*Ref*	*Ref*	*Ref*	*Ref*
Obese	2.32	1.01–5.64	0.053	2354.95	153.08–4556.82	0.036*	1.51	0.59–3.7	0.377	1.01	0.41–2.39	0.984	1.45	0.47–4.23	0.504
Race															
White	*Ref*	*Ref*	*Ref*	*Ref*	*Ref*	*Ref*	*Ref*	*Ref*	*Ref*	*Ref*	*Ref*	*Ref*	*Ref*	*Ref*	*Ref*
AIAN	4478298.89	0–NA	0.992	-468.5	-11068.87–10131.87	0.930	0	NA-	0.995	0	NA-	0.995	0	NA-	0.995
Asian	0.67	0.2–2.18	0.499	1142.27	-2008–4292.54	0.474	0.23	0.01–1.3	0.175	0.38	0.06–1.54	0.226	0.6	0.09–2.71	0.548
Black or AA	0.78	0.03–20.13	0.861	1647.5	-5894.22–9189.22	0.666	0	NA-	0.993	0	NA-	0.993	0	NA-	0.995
Other/Mixed	2.02	0.69–6.79	0.219	5624.78	2876.53–8373.02	< 0.0001*	1.08	0.32–3.26	0.894	0.8	0.24–2.37	0.698	1.21	0.29–4.38	0.782
Unknown	0.78	0.03–20.13	0.861	273	-7268.72–7814.72	0.943	0	NA-	0.993	0	NA-	0.993	0	NA-	0.995
Ethnicity															
Non-Hispanic	*Ref*	*Ref*	*Ref*	*Ref*	*Ref*	*Ref*	*Ref*	*Ref*	*Ref*	*Ref*	*Ref*	*Ref*	*Ref*	*Ref*	*Ref*
Hispanic/Latino	1.73	0.66–4.89	0.275	4240.13	1732.3–6747.96	0.001*	0.96	0.29–2.76	0.946	0.69	0.21–1.95	0.510	1.09	0.27–3.71	0.894
Smoking Status															
Never	*Ref*	*Ref*	*Ref*	*Ref*	*Ref*	*Ref*	*Ref*	*Ref*	*Ref*	*Ref*	*Ref*	*Ref*	*Ref*	*Ref*	*Ref*
Current	11833955.72	0–NA	0.991	455.79	-6118.95–7030.53	0.891	1.39	0.06–15.38	0.791	1.39	0.06–15.38	0.791	2.25	0.1–25.84	0.524
Former	1.64	0.58–5.11	0.368	-564.12	-3441.87–2313.63	0.698	0.74	0.19–2.36	0.635	1	0.29–3.01	0.995	2.81	0.72–10.42	0.122
Tumor (mm)															
MLD	1.05	1–1.12	0.084	154.05	20.55–287.55	0.024*	1.04	0.99–1.11	0.1412	1.03	0.98–1.09	0.297	1.03	0.96–1.09	0.403
BSC	1.02	0.96–1.09	0.524	140.7	-32.87–314.27	0.111	1.05	0.98–1.14	0.147	1.08	1.01–1.16	0.037*	1.04	0.96–1.13	0.313
Ant ext	1.12	1.01–1.26	0.0295*	198.55	-73.23–470.33	0.151	1.11	0.99–1.24	0.080	1.06	0.95–1.18	0.304	1.04	0.9–1.19	0.614
Post ext	1.04	0.97–1.12	0.291	217.57	29.77–405.37	0.024*	1.04	0.96–1.13	0.331	1.05	0.97–1.13	0.218	1.01	0.93–1.11	0.741

β – regression coefficient reported in minutes; AIAN – American Indian or Alaska Native; AA – African American; MLD – maximum linear dimension on magnetic resonance imaging; mm – millimeters; BSC – brainstem compression; Ant ext – anterior extension relative to the internal auditory canal; Post ext – posterior extension relative to the internal auditory canal; GTR – gross total resection; NTR – near total resection; STR – subtotal resection; Non-GTR defined as patients undergoing STR or NTR; EOR – extent of resection; LOS – hospital length of stay; FN – facial nerve; POD1 – postoperative day 1; BMI – body mass index (kg/m^2^); Non-obese defined as a BMI < 30 kg/m^2^; Obese defined as BMI 30 ≥ kg/m^2^; * indicate a significance *p* < 0.05

**Table 4:** Multivariate analysis of EOR, LOS, FN function

*N* = 116	EOR (ref GTR)			LOS			FN (POD1)^α^			FN (2–3 weeks postop)^β^			FN (latest)^δ^			
	OR	95% CI	*P*	Coefficient (β)	95% CI	*P*	OR	95% CI	*P*	OR	95% CI	*P*	OR		95% CI	*P*
Demographics																
Age	1.05	1.02–1.08	0.003*	79.84	9.6-150.1	0.026*	–	–	–	–	–	–	–	–		–
BMI																
Non-obese	–	–	–	*Ref*	*Ref*	*Ref*	–	–	–	–	–	–	–	–		–
Obese	–	–	–	905.74	-1380.6-3192.1	0.43	–	–	–	–	–	–	–	–		–
Race																
White	–	–	–	*Ref*	*Ref*	*Ref*	–	–	–	–	–	–	–	–		–
AIAN	–	–	–	1643.97	-8791.7-12079.6	0.76	–	–	–	–	–	–	–	–		–
Asian	–	–	–	1716.37	-1399.3-4832.0	0.28	–	–	–	–	–	–	–	–		–
Black or AA	–	–	–	1807.39	-6963.7-10578.5	0.68	–	–	–	–	–	–	–	–		–
Other/Mixed	–	–	–	3824.46	463.8-7185.1	0.026*	–	–	–	–	–	–	–	–		–
Ethnicity																
Non-Hispanic	–	–	–	*Ref*	*Ref*	*Ref*	–	–	–	–	–	–	–	–		–
Hispanic/Latino	–	–	–	1694.99	-1355.1-4745.1	0.009*	–	–	–	–	–	–	–	–		–
Tumor (mm)																
MLD	1.04	0.97–1.12	0.266	79.33	-101.12-259.79	0.39	–	–	–	0.99	0.91–1.06	0.92	1.02	0.94–1.09		0.61
BSC	0.99	0.88–1.11	0.855	56.80	-215.20-328.81	0.68	1.05	0.97–1.13	0.22	1.07	0.99–1.18	0.08	1.03	0.95–1.13		0.45
Ant ext	1.09	0.97–1.23	0.158	66.18	-217.88-350.24	0.64	1.10	0.98–1.24	0.11	1.04	0.93–1.18	0.48	–	–		–
Post ext	1.03	0.91–1.17	0.634	59.94	-249.21-369.08	0.70	–	–	–	–	–	–	–	–		–

Legend: AIAN – American Indian or Alaska Native; AA – African American; MLD – maximum linear dimension on magnetic resonance imaging; mm – millimeters; BSC – brainstem compression; Ant ext – anterior extension relative to the internal auditory canal; Post ext – posterior extension relative to the internal auditory canal; GTR – gross total resection; EOR – extent of resection; LOS – hospital length of stay; FN – facial nerve; POD1 – postoperative day 1; BMI – body mass index (kg/m^2^); Non-obese defined as a BMI < 30 kg/m^2^; Obese defined as BMI 30 ≥ kg/m^2^; *indicate a significance *p* < 0.05. ^α^FN POD1 had 27 events of HB III-VI with BSC and ant ext being the best fit model (lowest AIC); ^β^FN at 2–3 weeks postoperatively had 33 events of HB III-VI with MLD, BSC and ant ext being the best fit model (lowest AIC); ^δ^FN at latest follow up had 20 events of HB III-VI with MLD and BSC being the best fit model (lowest AIC)

### Hospital LOS outcomes

Univariate analysis demonstrated no significant association between BSC and anterior extension on LOS (*p* = 0.111 and *p* = 0.151, respectively), and no significant association in multivariate analysis (*p* > 0.05). Univariate analysis demonstrated a statistically significant relationship between posterior extension and LOS (β = 0.15 days; 95% CI 0.02–0.28 days, *p* = 0.024) (Table 3). After controlling for tumor dimensions and patient level characteristics, this association attenuated (β = 0.04 days, 95% CI -0.17–0.26, *p* = 0.22) (Table 4).

Age, ethnicity, race and BMI were each significantly associated with longer LOS in univariate analysis (age: β = 0.062 days, 95% CI 0.015–0.11 days, *p* = 0.011; ethnicity: β = 2.94 days, 95% CI 1.20–4.69 days, *p* = 0.001; mixed/other race: β = 3.91 days, 95% CI 2.00–5.82 days, *p* < 0.0001; BMI: β = 1.64 days, 95% CI 0.11–3.17 days, *p* = 0.036) (Table 3). In the fully adjusted multivariate model, older age (β = 0.056 days, *p* = 0.026), other/mixed race (β = 2.66 days, *p* = 0.026), and ethnicity (β = 1.18 days, *p* = 0.009) remained independently associated with increased LOS (Table 4).

### FN function outcomes

Median follow-up for FN function was 13.2 months (IQR 4.4–26.6; range = 0.3–81.4 months). Greater BSC was significantly associated with being in a poor HB grade (III–VI) at 2–3 weeks postoperatively in univariate analysis (OR = 1.08, 95% CI 1.01–1.16, *p* = 0.04) and failed to reach significance in multivariate analysis after controlling for tumor size and anterior extension (OR = 1.08, 95% CI 0.99–1.18, *p* = 0.08). Anterior extension, posterior extension, tumor size and age were not associated with FN function at any time points on univariate analysis (Table 3). On multivariate analysis, anterior extension, tumor size and BSC were not associated with FN function at any time points (Table 4).

## Discussion

In this study, we assessed how degree of BSC and anterior and posterior tumor extension relative to the IAC impact surgical outcomes in patients with large VS. Our analysis showed that greater anterior extension was significantly associated with a decreased likelihood of achieving GTR in univariate analysis, although this association lost significance after adjusting for covariates. Greater anterior extension was not significantly associated with postoperative FN function or LOS. Univariate analysis showed a significant relationship between greater posterior extension and longer LOS; however, on multivariate analysis this relationship was attenuated, and these initial results were likely confounded by age and patient level characteristics. Posterior extension did not demonstrate any significant correlation to EOR or postoperative FN function. Greater BSC was significantly associated with worse HB grade at 2–3 weeks postoperatively in univariate analysis, and failed to reach significance on multivariate analysis after controlling for tumor size and anterior extension. BSC was not correlated with any other outcome variables including EOR, immediate FN function postoperatively, FN function at the latest follow-up or LOS.

Age was the only predictor that was significantly associated with EOR and LOS in both univariate and multivariate analyses. LOS was also impacted by ethnicity and race in univariate and multivariate analyses.

### EOR

Anterior and posterior tumor extensions relative to IAC have been reported as possible predictors of EOR [10, 12, 13]. Our study revealed that greater anterior extension was associated with a decreased chance of GTR in univariate analysis. After controlling for BSC and posterior extension, this relationship remained significant, but lost significance after adjusting for tumor size and age. This finding contrasts with previous research. In an article published by Wong et al. [10] investigating 206 patients with VS, the authors reported that anterior tumor extension was a significant predictor of EOR in univariate analysis [10]. Another study by Manzoor et al. [12] found that greater anterior tumor extension was not only associated with STR but also with a higher likelihood of tumor regrowth and a shorter time to salvage therapy. Given the anatomy of the facial nerve which usually runs anterior to the tumor, greater anterior tumor extension results in greater displacement of the nerve, distorting the nerve-tumor interface and complicating gross tumor removal without damaging the nerve [12]. We hypothesize that this may lead to lesser EOR, as suggested by our analysis. EOR is complex and influenced by both tumor-specific characteristics (i.e., location, consistency, degree of adherence to adjacent neurovasculature) and patient-level factors (i.e., age, intraoperative technologist positioning). Patient-level factors may partially account for variability observed in EOR.

In contrast, posterior tumor extension was not identified as a significant predictor of EOR in our study. Although Wong et al. [10] reported a significant association between posterior extension and EOR, this relationship was not as robust as the one between anterior tumor extension and EOR, suggesting that posterior extension may be less critical in determining the EOR, possibly due to fewer critical adjacent structures.

Unlike previous reports that associate BSC with reduced GTR rates [10, 12, 14], our analysis did not find a significant relationship between BSC and EOR in both univariate and multivariate models. An article from 2024 reported a significant association between peduncular compression and EOR when analyzing all cases, yet notably, this association was not observed in their subgroup of patients with large tumors (> 3 cm) [14]. Taken together, these findings may suggest that in cases of large tumors with significant BSC, surgeons may have greater difficulty in achieving GTR due to a lack of a clear plane between the tumor and surrounding structures in the setting of tumor capsule adherence to the peduncle, brainstem, or FN [14, 15].

While many studies consistently demonstrate a significant correlation between larger tumor size and the likelihood of NTR/STR [11, 16–19], our study found that tumor size was not significantly associated with EOR (*p* = 0.084). This contradiction can be explained our inclusion criteria, which limited our study to tumors ≥ 3 cm. Grahnke et al. [13] included tumors ≥ 2.5 cm, reporting that patients with larger tumors were more prone to have STR, documenting a mean maximum extracanalicular tumor diameter of 3.68 cm in STR versus 3.26 cm in GTR [13]. It may be implied that once a certain metric threshold is reached, its impact on EOR may be negligible.

Older age at the time of surgery emerged as a significant predictor of EOR, increasing the likelihood of NTR/STR in both univariate and multivariate models, which is congruent with the literature, indicating that surgeons opt to be less aggressive in older patients compared to younger populations [10, 16, 17, 20]. At our center, younger patients may merit more aggressive tumor removal to avoid the need for more aggressive future treatment. On the other hand, we may be more conservative in elderly patients wherein the risk of needing further treatment with near or subtotal resection is very low.

The overwhelming percentage of approaches were translabyrinthine (TL) which reflects our institutional practice for large VS (> 3 cm) (Table 1). The TL approach offers an excellent anatomical view of the CPA and a direct approach to VS with high rates of FN preservation [21]. At our institution, the TL approach is performed in conjunction with a senior Neurosurgeon and senior Otolaryngologist present, thereby potentially limiting generalizability to other institutions. Further studies incorporating different surgical approaches may help further delineate the interaction between surgical approach, tumor anatomy and EOR.

### LOS

Several demographic factors demonstrated associations with LOS. Similar to other studies [22, 23], age was a significant predictor of longer LOS in both univariate and multivariate models. In the multivariate model, every 1-year increase in age was associated with an additional ~ 0.06 days (~ 80 min) longer LOS. Similar to findings from O’Connell et al. [24], patients with obesity had a significantly longer LOS by ~ 1.64 days in univariate analysis, but after controlling for age, ethnicity, race, and tumor dimensions this relationship was non-significant. Previous studies have evaluated how frailty, as measured by the modified frailty index, independently influences discharge disposition and LOS beyond age alone, with higher frailty associated with increased LOS and perioperative complications in VS patients [25, 26]. Frailty could not be evaluated in the present study due to absence of standardized frailty metrics, but remains an important patient-level factor that should be incorporated into surgical risk stratification in future studies.

Race and ethnicity were significant predictors of LOS in univariate and multivariate analyses, with other/mixed race patients having a longer LOS by ~ 2.65 days compared to white patients, and ~ 1.18 days compared to non-Hispanic patients. A study by Cutri et al. [27] examined how racial and socioeconomic status influence VS presentation and found that Hispanic/Latino individuals were significantly more likely to have worse hearing status and larger tumor size compared to white patients. Studies have shown that individuals with lower socioeconomic status face barriers to accessing healthcare, which may delay VS diagnosis and result in more complex pathology and larger tumor size, resulting in longer LOS [28–30].

Tumor size—and its effect on LOS—has mixed findings in the literature. Vorasubin et al. [22] found no effect of tumor size or surgical approach on LOS in a cohort of 288 patients with VS, whereas Visagan et al. [31] did, specifically for tumors between 31 and 40 mm. Another study by Sanna et al. [32] found a much higher incidence of postoperative complications in tumors > 30 mm, resulting in longer LOS compared to patients with smaller tumors [32]. Our findings align with Vorasubin et al. [22] whereby no significant association between tumor size and LOS was found.

It has been reported that females may experience a longer LOS compared to men due to a higher prevalence of vertigo and dizziness [22]; however, we did not find any significant relationship between sex and LOS. On average, males had a longer LOS compared to females by ~ 0.82 days (~ 19 h and 38 min), but this relationship was not statistically significant.

Taken together our findings suggest that age is the most robust predictor of LOS, and that race, ethnicity and obesity may reflect underlying disparities in access to care and disease severity at presentation. The lack of robust association between tumor morphology and LOS indicate patient specific characteristics, such as frailty may more directly drive LOS.

### FN function

Numerous studies have examined various preoperative predictors of postoperative FNO. Larger tumor size has been consistently shown to carry a higher risk of FN dysfunction, both immediately and at long-term postoperative follow-ups [11, 33–35]. This may be because larger tumors have a broader nerve-capsule interface and thus a higher likelihood of stretch injury [11, 36, 37]. For instance, in a study of 400 patients with VS > 2.5 cm, Schwartz et al. [38] reported a significant difference in mean tumor size in patients with poor FNO compared to those with good FNO (3.4 vs. 3.1 cm). However, we found that tumor size was not a significant predictor of FNO at any postoperative time point. These contradictory findings are likely due to our inclusion of only large VS > 3 cm, and our surgical philosophy prioritizing functional preservation.

It is not known whether it is purely tumor size or the specific anatomical direction of tumor growth that determines FN injury risk. Anterior tumor extension is a particularly critical factor, since in most tumors, the FN lies anteriorly; therefore, as the tumor grows anteriorly, it displaces, stretches, and distorts the nerve, making it vulnerable to injury during dissection [10, 11]. In our study, anterior extension was not found to be significant predictor of FNO at any timepoint, which contradicts other studies who reported a significant correlation between FNO and anterior extension [10, 11, 13, 33, 39, 40]. For instance, Grahnke et al. [13] reported a 16% increased likelihood of a worse HB score for every 1 mm increase in anterior tumor extension. The inconsistency with the literature could again be influenced by our surgical emphasis on functional preservation.

Other studies have reported no correlation between posterior tumor extension and worse FNO, findings that align with our study [13, 33, 40]. Therefore, tumors that predominantly extend posteriorly relative to the IAC could be considered lower FN risk [13]. This is supported by Hobson et al. [33], whereby a greater posterior to anterior extension ratio was reported in patients with good FNO compared to those with poor outcome postoperatively.

The impact of BSC on postoperative FNO has yielded variable results across different studies [10–14]. In our cohort, BSC approached a significant relationship with worse FNO immediately post-surgery. Notably, at 2–3 weeks postoperatively, BSC was found to be associated with poor HB grade in univariate analysis but failed to reach significance in multivariate analysis after controlling for tumor size and anterior extension (*p* = 0.08). BSC was not associated with FNO at the latest follow-up (median 13.2 months). This aligns with Perkins et al. [11], who observed that at 2–3 weeks postoperatively, BSC, along with larger tumor volume and greater ventral extension, was associated with poor FNO. However, this significance was lost at 1-year follow-up, suggesting a potential transient impact of BSC on FNO, a finding similar to Perkins et al. [11]. Moreover, Tai et al. [14] reported that despite being significantly associated in medium-sized tumors (15–30 mm), peduncular compression was not observed to be related with unfavorable FNO in larger tumors (≥ 30 mm) immediately postoperatively and at last follow-up. Peritumor brainstem edema in the CPA occurs in approximately half of patients with large VS, and is associated with worse preoperative hearing, lower rates of GTR and higher rates of postoperative vestibular dysfunction [41]. While this study was focused on discrete linear tumor measurements, anticipated surgical challenges corresponding to brainstem edema should inform preoperative counseling with respect to EOR goals and postoperative recovery expectations.

## Limitations

This is a retrospective study that lacks a control group thereby limiting direct comparisons. Certain predictors and outcomes were subject to small sample sizes, limiting robust conclusions. For example, 37 of 116 patients had no record of long-term postoperative FN function. This could underestimate post-operative FNO and lead to attrition bias. Lastly, this a single institutional study involving two surgeons, limiting the generalizability of this study. However, this is arguably a strength of the study, as removing variation in surgical practice allows for isolated study of radiographic features as analyzed in this study. Additionally, measures of frailty were not available in the medical records and therefore could not be assessed as a covariate in our models. Given the association between frailty, age, and surgical morbidity, future studies should incorporate standardized frailty metrics to better characterize patient risk factors to guide surgical decision-making. Lastly, EOR is based on intraoperative subjectivity of the senior neurosurgeon, thereby potentially limiting the generalizability of these results.

## Conclusion

Greater BSC may be associated with transient short-term FNO. EOR and LOS do not appear to be influenced by anterior or posterior extension, respectively, but rather, appear to be confounded by age. Tumor morphology may play a role in outcome prediction and could inform surgical decision-making. Surgical planning should integrate radiographic and patient-level characteristics to optimize management of large VS.

## Data Availability

No datasets were generated or analysed during the current study.

## References

[1] Yu Y, Song G, Zhao Y, Liang J, Liu Q (2023) Prediction of Vestibular Schwannoma Surgical Outcome Using Deep Neural Network. World Neurosurg Aug 176:e60–e67. 10.1016/j.wneu.2023.03.090 10.1016/j.wneu.2023.03.090 36966911

[2] Starnoni D, Giammattei L, Cossu G et al (2020) Surgical management for large vestibular schwannomas: a systematic review, meta-analysis, and consensus statement on behalf of the EANS skull base section. Acta Neurochir (Wien) Nov 162(11):2595–2617. 10.1007/s00701-020-04491-7 10.1007/s00701-020-04491-7 PMC7550309 32728903

[3] Gal TJ, Shinn J, Huang B (2010) Current epidemiology and management trends in acoustic neuroma. Otolaryngol Head Neck Surg May 142(5):677–681. 10.1016/j.otohns.2010.01.037 10.1016/j.otohns.2010.01.037 20416455

[4] Deen HG, Ebersold MJ, Harner SG et al (Aug 1996) Conservative management of acoustic neuroma: an outcome study. Neurosurgery 39(2):260-4; discussion 264-6. 10.1097/00006123-199608000-00005 10.1097/00006123-199608000-00005 8832662

[5] Misra BK, Purandare HR, Ved RS, Bagdia AA, Mare PB (2009) Current treatment strategy in the management of vestibular schwannoma. Neurol India May-Jun 57(3):257–263. 10.4103/0028-3886.53263 10.4103/0028-3886.53263 19587464

[6] Gupta VK, Thakker A, Gupta KK (2020) Vestibular Schwannoma: What We Know and Where We are Heading. Head Neck Pathol Dec 14(4):1058–1066. 10.1007/s12105-020-01155-x 10.1007/s12105-020-01155-x PMC7669921 32232723

[7] Khan NR, Elarjani T, Jamshidi AM et al (2022) Microsurgical Management of Vestibular Schwannoma (Acoustic Neuroma): Facial Nerve Outcomes, Radiographic Analysis, Complications, and Long-Term Follow-Up in a Series of 420 Surgeries. World Neurosurg Dec 168:e297–e308. 10.1016/j.wneu.2022.09.125 10.1016/j.wneu.2022.09.125 36198364

[8] Falcioni M, Fois P, Taibah A, Sanna M (2011) Facial nerve function after vestibular schwannoma surgery. J Neurosurg Oct 115(4):820–826. 10.3171/2011.5.Jns101597 10.3171/2011.5.JNS101597 21682562

[9] Monfared A, Corrales CE, Theodosopoulos PV et al (2016) Facial Nerve Outcome and Tumor Control Rate as a Function of Degree of Resection in Treatment of Large Acoustic Neuromas: Preliminary Report of the Acoustic Neuroma Subtotal Resection Study (ANSRS). Neurosurgery Aug 79(2):194–203. 10.1227/neu.0000000000001162 10.1227/NEU.0000000000001162 26645964

[10] Wong RH, Copeland WR, Jacob JT et al (2017) Anterior Extension of Tumor is as Important as Tumor Size to Facial Nerve Outcome and Extent of Resection for Vestibular Schwannomas. J Neurol Surg B Skull Base Dec 78(6):473–480. 10.1055/s-0037-1604331 10.1055/s-0037-1604331 PMC5680033 29134166

[11] Perkins EL, Manzoor NF, Totten DJ et al (2021) The Influence of Extent of Resection and Tumor Morphology on Facial Nerve Outcomes Following Vestibular Schwannoma Surgery. Otol Neurotol Oct 1(9):e1346–e1352. 10.1097/mao.0000000000003253 10.1097/MAO.0000000000003253 34238899

[12] Manzoor NF, Nassiri AM, Sherry AD et al (2022) Predictors of Recurrence After Sub-total or Near-total Resection of Vestibular Schwannoma: Importance of Tumor Volume and Ventral Extension. Otol Neurotol Jun 1(5):594–602. 10.1097/mao.0000000000003477 10.1097/MAO.0000000000003477 35184072

[13] Grahnke K, Garst JR, Martin B, Leonetti JP, Anderson DE (2017) Prognostic Indices for Predicting Facial Nerve Outcome following the Resection of Large Acoustic Neuromas. J Neurol Surg B Skull Base Dec 78(6):454–460. 10.1055/s-0037-1604077 10.1055/s-0037-1604077 PMC5680026 29134163

[14] Tai A, Kim J, Croci D et al (2024) Significant tumor compression of the middle cerebellar peduncle is associated with worse facial nerve outcomes and lower extent of resection in surgery for medium-sized vestibular schwannomas - A radiographic analysis of a case series. Clin Neurol Neurosurg Jan 236:108114. 10.1016/j.clineuro.2024.108114 10.1016/j.clineuro.2024.108114 38232608

[15] Jung GS, Montibeller GR, Fraga GS, Rohde TDS, Ramina R (2021) Facial Nerve Adherence in Vestibular Schwannomas: Classification and Radiological Predictors. J Neurol Surg B Skull Base Aug 82(4):456–460. 10.1055/s-0040-1713103 10.1055/s-0040-1713103 PMC9100449 35573922

[16] Sughrue ME, Kaur R, Rutkowski MJ et al (2011) Extent of resection and the long-term durability of vestibular schwannoma surgery. J Neurosurg May 114(5):1218–1223. 10.3171/2010.11.Jns10257 10.3171/2010.11.JNS10257 21250800

[17] Macielak RJ, Lohse CM, Wallerius KP et al (2022) Predicting Extent of Microsurgical Resection of Sporadic Vestibular Schwannoma. Otol Neurotol Sep 1(8):950–955. 10.1097/mao.0000000000003593 10.1097/MAO.0000000000003593 35941666

[18] Noureldine MHA, Aum D, Piper K et al (2020) Value of the Petromeatal Angle in Predicting Outcome of Translabyrinthine Resection of Vestibular Schwannomas. Oper Neurosurg Sep 15(4):E370–e378. 10.1093/ons/opaa109 10.1093/ons/opaa109 32348494

[19] Nandoliya KR, Khazanchi R, Winterhalter EJ et al (2025) Extent of resection and progression-free survival in vestibular schwannoma: a volumetric analysis. J Neurosurg Jan 1(1):230–238. 10.3171/2024.4.Jns24157 10.3171/2024.4.JNS24157 39094197

[20] Wang SS, Machetanz K, Ebner F, Naros G, Tatagiba M (2023) Association of extent of resection on recurrence-free survival and functional outcome in vestibular schwannoma of the elderly. Front Oncol 13:1153698. 10.3389/fonc.2023.1153698 37342182 10.3389/fonc.2023.1153698 PMC10277928

[21] Mamikoglu B, Wiet RJ, Esquivel CR (2002) Translabyrinthine Approach for the Management of Large and Giant Vestibular Schwannomas. Otology Neurotology 23(2):224–227 11875354 10.1097/00129492-200203000-00020

[22] Vorasubin N, Alexander TH, Mastrodimos B, Cueva RA (2018) Factors That Affect Length of Hospital Stay After Vestibular Schwannoma Surgery. Otol Neurotol Oct 39(9):1203–1209. 10.1097/mao.0000000000001960 10.1097/MAO.0000000000001960 30199503

[23] Sylvester MJ, Shastri DN, Patel VM et al (2017) Outcomes of Vestibular Schwannoma Surgery among the Elderly. Otolaryngol Head Neck Surg Jan 156(1):166–172. 10.1177/0194599816677522 10.1177/0194599816677522 28045630

[24] O’Connell BP, Rizk HG, Stevens SM, Nguyen SA, Meyer TA (2015) The Relation between Obesity and Hospital Length of Stay after Elective Lateral Skull Base Surgery: An Analysis of the American College of Surgeons National Surgical Quality Improvement Program. ORL J Otorhinolaryngol Relat Spec 77(5):294–301. 10.1159/000435786 26360829 10.1159/000435786

[25] Tang OY, Bajaj AI, Zhao K et al (2022) Association of Patient Frailty With Vestibular Schwannoma Resection Outcomes and Machine Learning Development of a Vestibular Schwannoma Risk Stratification Score. Neurosurgery Aug 1(2):312–321. 10.1227/neu.0000000000001998 10.1227/neu.0000000000001998 35411872

[26] Steele JL, Ewer NJ, Smith HJ et al (2025) Frailty, surgical time, and surgical complications increase length of stay following large vestibular schwannoma resection. Otolaryngol Head Neck Surg Oct 31. 10.1002/ohn.70063 10.1002/ohn.70063 PMC12860176 41169251

[27] Cutri RM, Lin J, Wilson ML, Doherty JK, Pan DW (2024) Disparities in Sporadic Vestibular Schwannoma Initial Presentation Between a Public Safety Net Hospital and Tertiary Academic Medical Center at the Same Zip Code 2010 to 2020. Ann Otol Rhinol Laryngol Jun 133(6):605–612. 10.1177/00034894241241201 10.1177/00034894241241201 38517145

[28] Weissman JS, Stern R, Fielding SL, Epstein AM (1991) Delayed access to health care: risk factors, reasons, and consequences. Ann Intern Med Feb 15(4):325–331. 10.7326/0003-4819-114-4-325 10.7326/0003-4819-114-4-325 1899012

[29] Ellsperman SE, Bellile E, Fryatt R et al (2023) The Impact of Social Determinants of Health on Vestibular Schwannoma Management: A Single Institution Review. Otol Neurotol Jun 1(5):507–512. 10.1097/mao.0000000000003883 10.1097/MAO.0000000000003883 37167450

[30] Dreyer V, Harris P, Daggubati L (2025) Race and Socioeconomic Status in the Management of Vestibular Schwannomas: Interactions and Trends Over the Past 20 Years. J Neurol Surg B Skull Base 2025/02/07(S 01):P468. 10.1055/s-0045-1803946

[31] Visagan R, Hall A, Bradford R, Khalil S, Saeed SR (2019) Is There a Difference in Hospital Stay between Patients undergoing Translabyrinthine or Retrosigmoid Surgery for Vestibular Schwannoma Stratified by Tumor Size? J Neurol Surg B Skull Base Jun 80(3):310–315. 10.1055/s-0038-1668541 10.1055/s-0038-1668541 PMC6534737 31143576

[32] Sanna M, Taibah A, Russo A, Falcioni M, Agarwal M (2004) Perioperative complications in acoustic neuroma (vestibular schwannoma) surgery. Otol Neurotol May 25(3):379–386. 10.1097/00129492-200405000-00029 10.1097/00129492-200405000-00029 15129121

[33] Hobson CE, Saliba J, Vorasubin N, Lyles RH, Mastrodimos B, Cueva RA (2022) Vestibular Schwannoma Cerebellopontine Angle Position Impacts Facial Outcome. Laryngoscope May 132(5):1093–1098. 10.1002/lary.29922 10.1002/lary.29922 34704617

[34] Ren Y, MacDonald BV, Tawfik KO, Schwartz MS, Friedman RA (2021) Clinical Predictors of Facial Nerve Outcomes After Surgical Resection of Vestibular Schwannoma. Otolaryngol Head Neck Surg May 164(5):1085–1093. 10.1177/0194599820961389 10.1177/0194599820961389 33048002

[35] Troude L, Boucekine M, Montava M, Lavieille JP, Régis JM, Roche PH (2019) Predictive Factors of Early Postoperative and Long-Term Facial Nerve Function After Large Vestibular Schwannoma Surgery. World Neurosurg Jul 127:e599–e608. 10.1016/j.wneu.2019.03.218 10.1016/j.wneu.2019.03.218 30930324

[36] Gurgel RK, Theodosopoulos PV, Jackler RK (2012) Subtotal/near-total treatment of vestibular schwannomas. Curr Opin Otolaryngol Head Neck Surg Oct 20(5):380–384. 10.1097/MOO.0b013e328357b220 10.1097/MOO.0b013e328357b220 22954813

[37] Bloch O, Sughrue ME, Kaur R et al (2011) Factors associated with preservation of facial nerve function after surgical resection of vestibular schwannoma. J Neurooncol Apr 102(2):281–286. 10.1007/s11060-010-0315-5 10.1007/s11060-010-0315-5 PMC3052445 20694574

[38] Schwartz MS, Kari E, Strickland BM et al (2013) Evaluation of the increased use of partial resection of large vestibular schwanommas: facial nerve outcomes and recurrence/regrowth rates. Otol Neurotol Oct 34(8):1456–1464. 10.1097/MAO.0b013e3182976552 10.1097/MAO.0b013e3182976552 23928516

[39] Gerganov VM, Klinge PM, Nouri M, Stieglitz L, Samii M, Samii A (2009) Prognostic clinical and radiological parameters for immediate facial nerve function following vestibular schwannoma surgery. Acta Neurochir (Wien) Jun 151(6):581–587 discussion 587. 10.1007/s00701-009-0288-3 10.1007/s00701-009-0288-3 19337682

[40] Sharma M, Sonig A, Ambekar S, Nanda A (2013) Radiological and Clinical Factors Predicting the Facial Nerve Outcome following Retrosigmoid Approach for Large Vestibular Schwannomas (VSs). J Neurol Surg B Skull Base Oct 74(5):317–323. 10.1055/s-0033-1349060 10.1055/s-0033-1349060 PMC3788334 24436931

[41] Ung TH, Freeman L, Hirt L et al (2023) Surgical outcomes in large vestibular schwannomas: should cerebellopontine edema be considered in the grading systems? Acta Neurochir (Wien) Jul 165(7):1749–1755. 10.1007/s00701-023-05627-1 10.1007/s00701-023-05627-1 37204532

